# miR-382-5p promotes cell invasion in hepatocellular carcinoma by targeting PTEN to activate PI3K/Akt signaling pathway

**DOI:** 10.1186/s12957-022-02638-7

**Published:** 2022-06-02

**Authors:** Bo Lv, Xianzhuo Liu, Xinfeng Zhu, Min Huang

**Affiliations:** 1 Department of Hepatobiliary and Pancreatic Surgery, Wuhan Central Hospital, Tongji Medical College, Huazhong University of Science and Technology, 430014 Wuhan, China; 2 Department of Emergency, Wuhan Central Hospital, Tongji Medical College, Huazhong University of Science and Technology, 430014 Wuhan, China; 3 School of Information Engineering, Wuhan College, 430212 Wuhan, China

**Keywords:** miR-382-5p, Hepatocellular carcinoma, PTEN, PI3K/Akt, Invasion

## Abstract

**Purpose:**

This study aimed at investigating miR-382-5p expression in tissues and cell lines with hepatocellular carcinoma (HCC), its effects on the invasion of HCC cells, and related mechanisms.

**Methods:**

miR-382-5p expression in HCC tissues, adjacent tissues, cell lines of normal hepatic cells, and HCC cells were detected by qRT-PCR, indicating its upregulation or downregulation in HCC cell lines (Hep3B and HCCLM3). The effect of miR-382-5p on cell invasion was observed by the Transwell experiment. The targeting relationship of miR-382-5p and the phosphatase and tensin homolog (PTEN) was analyzed using bioinformatics tools and the luciferase reporter gene assay. The correlation between miR-382-5p and PTEN was analyzed with Spearman correlation analysis. PTEN expression was observed after upregulation and downregulation of miR-382-5p expression. The effect of miR-382-5p on the expression of key proteins in PI3K/Akt signaling pathway was determined by Western blot.

**Results:**

miR-382-5p expression was upregulated in both HCC tissues and cell lines (both *P*<0.05). Upregulation or downregulation of miR-382-5p significantly promoted or inhibited the invasion of cell lines, Hep3B, and HCCLM3. The luciferase reporter gene assay confirmed that PTEN is a target of miR-382-5p. The expressions of miR-382-5p and PTEN were negatively correlated (*r*=−0.742, *P*<0.001). Upregulation of PTEN expression by plasmid transfection can reverse the invasive effect of miR-382-5p on HCC cells. Upregulation of miR-382-5p can activate PI3K/Akt signaling pathway, and downregulation of miR-382-5p can inhibit PI3K/Akt signaling pathway.

**Conclusions:**

miR-382-5p can activate the PI3K/Akt signaling pathway by targeting PTEN and promote HCC cell invasion.

## Introduction

Hepatocellular carcinoma (HCC) is a common malignancy, ranking third in global cancer mortality [1]. It is closely related to hepatitis B virus, while China is the prevalence area of hepatitis B. So the incidence of HCC is high in China [2]. Despite recent advances in the diagnosis and treatment strategies for HCC, the prognosis of patients with HCC remains poor due to metastasis and relapse [3, 4]. Identifying biomarkers associated with HCC progression is particularly important for early diagnosis of HCC, discovery of new therapeutic targets, and improvement of prognosis [5]. microRNA (miRNA) is a small non-coding RNA molecule that can regulate target gene expression by the post-transcriptional mechanism [6]. A large number of studies have shown that as a tumor promoter or inhibitor, miRNA regulates the biological processes of tumor cells, including cell proliferation, cell cycle, invasion, migration, autophagy, and cell senescence [7–10]. microRNA-382-5p is the newly identified miRNA molecule that is upregulated in breast cancer and promotes the development and progression of breast cancer [11]. Studies have shown that microRNA-382-5P is upregulated in leukemia [12], and it promotes the progression of leukemia by inhibiting phosphatase and tensin homolog (PTEN) expression. However, its role in HCC remains completely unclear.

PTEN is a widely well-studied tumor suppressor molecule with significantly decreased PTEN expression in glioblastoma, breast cancer, endometrial cancer, ovarian cancer, lung cancer, prostate cancer, colorectal cancer, and HCC [13–15]. Studies have shown that many different miRNAs in humans are related to the regulation of PTEN. For example, microRNA-19a-3p can regulate PTEN expression to promote the progression of cancers [16]. However, the relationship between PTEN and microRNA-382-5p in HCC remains unclear. In this study, we explored the expression of miR-382-5p and PTEN in tissues and cell lines with HCC, and the potential molecular mechanisms of miR-382-5p in the occurrence and progression of HCC.

## Methods and materials

### Tissue samples

In this study, specimens of HCC tissues and adjacent tissues were collected from 30 patients who underwent HCC excision in General Surgery in our hospital from January 2016 to December 2020. Of all the patients, there were 19 males and 11 females, ranging from 42 to 67 years old, with the mean age of 52.9 ± 12.3 years old. There were 14 cases of TNM I, 10 cases of stage II, 5 cases of stage III, and 1 case of stage IVa. All the specimens collected were kept in the refrigerator at −80°C.

### Culture and transfection of cells

HCC cell lines (Hep3B, SMMC-7721, MHCC97-L, and HCCLM3) and normal human hepatocyte cell line L02 were presented by the General Surgery Laboratory of Tongji Hospital Affiliated to Tongji Medical College of Huazhong University of Science and Technology. All the cell lines were cultured in Dulbecco’s modified Eagle’s medium (DMEM; Gibco, Grand Island, NY, USA) containing 10% fetal bovine serum (FBS, Gibco) and 1% penicillin-streptomycin (Sigma, St-Louis, MO, USA) and in a humidified incubator containing 5% CO_2_ at 37°C. miR-382-5p mimics, negative control sequences (Scramble), and miR-382-5p inhibit were purchased from the Ruibo Biotechnology, Guangzhou, China. PTEN overexpression plasmid was synthesized by the Shanghai GenePharma Biotechnology Corporation. Lipofectamine^TM^3000 transfection reagent was purchased from Invitrogen (Carlsbad, CA, USA). PTEN primary antibody (Cargo No. 9188, Cell Signaling Technology, USA), GAPDH (cargo number 5174, Cell Signaling Technology, USA), p-PI3K primary antibody (Cargo No. 4228, Cell Signaling Technology, USA), PI3K primary antibody (Cargo No. 4255S, Cell Signaling Technology, USA), Akt primary antibody (Cargo No. 4691, Cell Signaling Technology, USA), and p-Akt (Cargo No. 4060, Cell Signaling Technology, USA) were adopted in this study.

### Cell grouping

HCC cell lines (Hep3B and HCCLM3) were transfected with miR-382-5p mimics, scramble, and miR-382-5p inhibit, respectively. Then, they were divided into miR-382-5p overexpression (miR-382-5p mimics) group, negative control (scramble) group, and miR-382-5p downregulated expression (miR-382-5p inhibit) group, followed by PCR, Transwell and Western blot experiments.

### qRT-PCR

RNA was extracted from tissues and cells with Trizol (Invitrogen, USA), following the manufacturer’s procedures. Reverse transcription was performed with TIAN Script RT kit (Tiangen Biotech, Beijing, China). qPCR was performed with the TaqMan Human MiRNA assay kit (Genecopoeia) and the SYBR Premix Ex TaqTM kit (TaKaRa, Shiga, Japan). U6 was taken as the internal reference, with the primer sequences, including forward: 5′-CTCGCTTCGGCAGCACA-3′, reverse: 5′-AACGCTTCACGAATTTGCGT-3. Expression levels were tested with 2^-ΔΔ^ Ct, which was quantified and repeated in triplicate. qPCR temperature cycles were performed as follows: 50 °C for 2 min and 95 °C for 10 min; 45 cycles at 95 °C for 15 s and 60 °C for 1 min.

### Cell invasion assay

Transwell chamber (Millipore, Burlington, MA, USA) was adopted for cell invasion assays. Hep3B and HCCLM3 cell lines (2×10^4^) in the upper chamber were cultured with serum-free DMEM, and the lower chamber was filled with 10% serum-containing DMEM. Hep3B and HCCLM3 cells (2 × 10^4^) were inoculated on Matrigel-coated membrane inserts. Then, the chamber was placed on the cell culture plate and incubated at 37°C for 24 h. Subsequently, the cells passed through the migration pore membrane were fixed in 4% paraformaldehyde for 10 min and then stained with 0.1% crystal violet for 20 min. Finally, the migrating cells were counted under a microscope.

### Western blot

Proteins in cell lines and tissues with HCC were extracted with RIPA buffer, and the concentrations were measured with the BCA kit. Proteins were then electrophoresed by 10% SDS-PAGE electrophoresis and transferred onto the PVDF membrane (Bio-Rad, Hercules, CA, USA). Then, the membrane was blocked in 5% skim milk and incubated with the specific primary antibody at 4 °C overnight, including PTEN primary antibody (1:300), Akt primary antibody (1:300), p-Akt primary antibody (1:200), p-PI3K primary antibody (1:300), PI3K primary antibody (1:300), and GAPDH primary antibody (1:500). The membranes were incubated with the secondary antibody (1:500) at room temperature for 1 h and exposed with the ECL developer in a darkroom.

### Luciferase reporter gene assay

Wild-type (WT) or mutant (MUT) plasmid, 3′UTR, of PTEN mRNA was synthesized and inserted downstream of pEZX-MT06 vector (Genecopoeia). PTEN-3'UTR-WT and PTEN-3'UTR-MUT of Hep3B cells were transfected with miR-382-5p mimics or scramble, respectively. The cells were collected after 48 h, and the luciferase activity was quantified with the Luc-PairTM Duo-luciferase assay kit.

### Statistical analysis

Data were expressed as mean ± standard deviation and analyzed with GraphPad Prism 7.0 software. All experiments were repeated at least 3 times. *t* test (two groups) and one-way ANOVA (multiple groups) were performed to analyze the differences. Spearman correlation analysis was conducted to analyze the correlation between miR-382-5p and PTEN expression. *P* <0.05 indicated a statistically significant difference.

## Results

### Comparison of miR-382-5p expression in tissues and cell lines with HCC

**Fig. 1:**
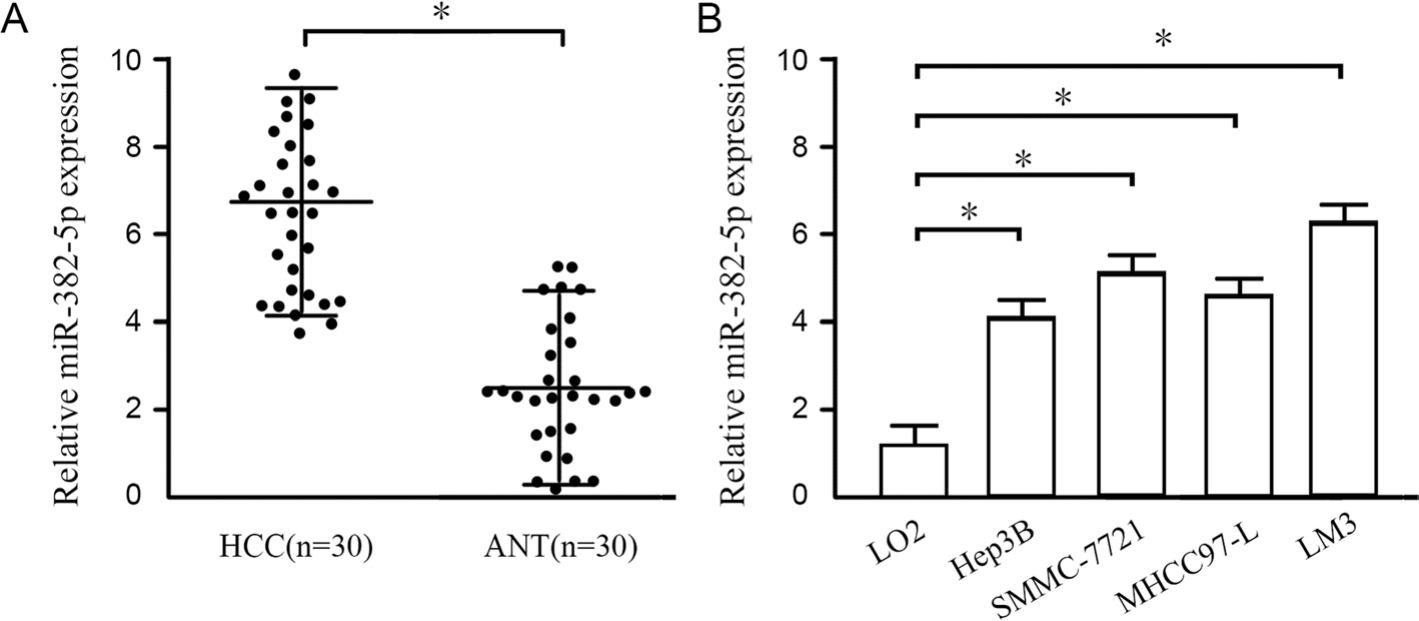
Comparison of miR-382-5p expression in HCC tissues and cell lines. **A** miR-382-5p expression in HCC tissues and adjacent tissues. **B** miR-382-5p expression in HCC cell lines and normal hepatic cell line LO2; Note: HCC: HCC tissues, ANY: adjacent normal tissues

miR-382-5p expression in 30 pairs of HCC tissues and adjacent normal hepatic tissues was detected by qRT-PCR, which was higher in HCC tissues than that in adjacent normal hepatic tissues (*P*<0.001), as shown in Fig. 1A. miR-382-5p expression was significantly upregulated in HCC cell lines (Hep3B, SMMC-7721, MHCC97-L, and HCCLM3) compared with that in normal hepatic tissues (L02) (*P*<0.05), as shown in Fig. 1B.

### Effect of miR-382-5p on cell invasion in HCC cell lines

**Fig. 2:**
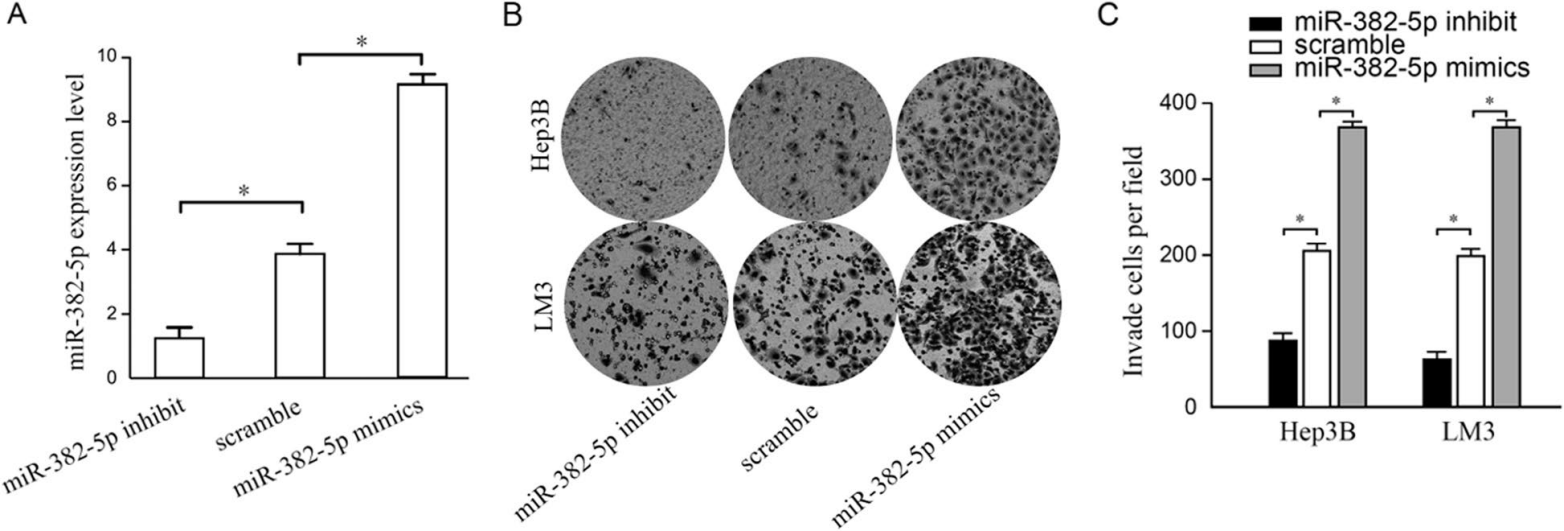
Effect of miR-382-5p on cell invasion in HCC cell lines. **A** Transfection efficiency. **B** Transwell experiment. **C** Comparison of invaded cells; *P <0.05

HCC cell lines (Hep3B and HCCLM3) were transfected with miR-382-5p mimics, scramble, and miR-382-5p inhibit, respectively. Higher expression of miR-382-5p were found in the miR-382-5p mimics group by qRT-PCR, compared with that in the scramble group (*P* <0.05). miR-382-5p expression was lower in the miR-382-5p inhibit group than that in the scramble group (*P*<0.05). The results indicated a successful transfection and the follow-up experiments were feasible, as shown in Fig. 2A.

Transwell experiments showed that the miR-382-5p mimics group had more invaded cells than that in the scramble group (*P*<0.05) and the miR-382-5p inhibit group had less invaded cells than that in the scramble group (*P* <0.05). All the results above indicated that upregulation of miR-382-5p expression can promote HCC cell invasion, and otherwise, the downregulation of miR-382-5p expression can inhibit HCC cell invasion, as shown in Fig. 2B.

### Target gene of miR-382-5p was PTEN

**Fig. 3:**
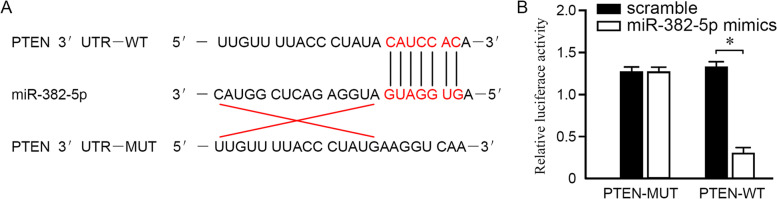
Target gene of miR-382-5p was PTEN. **A** TargetScan predicted a complementary sequence with miR-382-5p in the 3'UTR of PTEN mRNA. **B** Duo-luciferase assay confirmed the target gene of miR-382-5p as PTEN

The potential target gene was predicted for miR-382-5ps with the TargetScan (http://www.targetscan.org). A complementary sequence with miR-382-5p was found in the 3'UTR of PTEN mRNA, namely binding sites, as shown in Fig. 3A. Duo-luciferase assays showed that miR-382-5p mimics significantly inhibited the luciferase activity in PTEN-3′UTR-WT, but not luciferase activity in PTEN-3′UTR-MUT (*P* <0.05), as shown in Fig. 3B.

### Correlation between PTEN expression and miR-382-5p

**Fig. 4:**
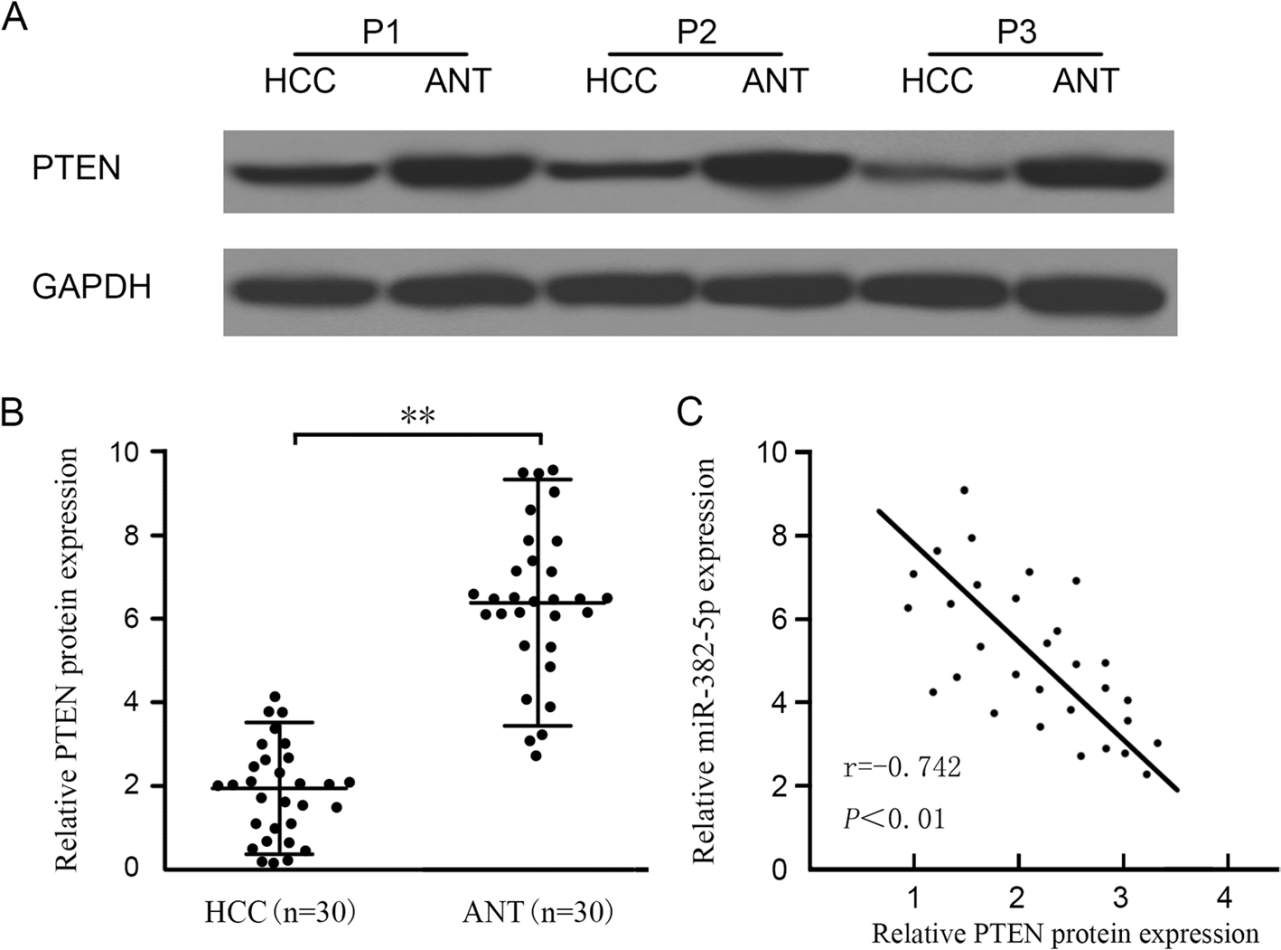
Correlation of PTEN expression and miR-382-5p. **A** Western blot for PTEN expression in HCC tissues and adjacent tissues (3 pairs); **B** PTEN expression in HCC tissues and adjacent tissues (*n*=30). **C** Spearman correlation analysis showed that miR-382-5p is negatively associated with PTEN expression

Western blot results showed that PTEN expression was lower in HCC tissues than that in adjacent tissues, as shown in Fig. 4A. It was found that among 30 pairs of HCC tissues and adjacent tissues, PTEN expression was lower in HCC tissues than that in adjacent tissues, as shown in Fig. 4B. Spearman correlation analysis showed a negative correlation between the expression levels of miR-382-5p and PTEN (*r*= −0.742, *P*<0.01), as shown in Fig. 4C.

### Regulatory relationship between miR-382-5p and PTEN in HCC cell lines

**Fig. 5:**
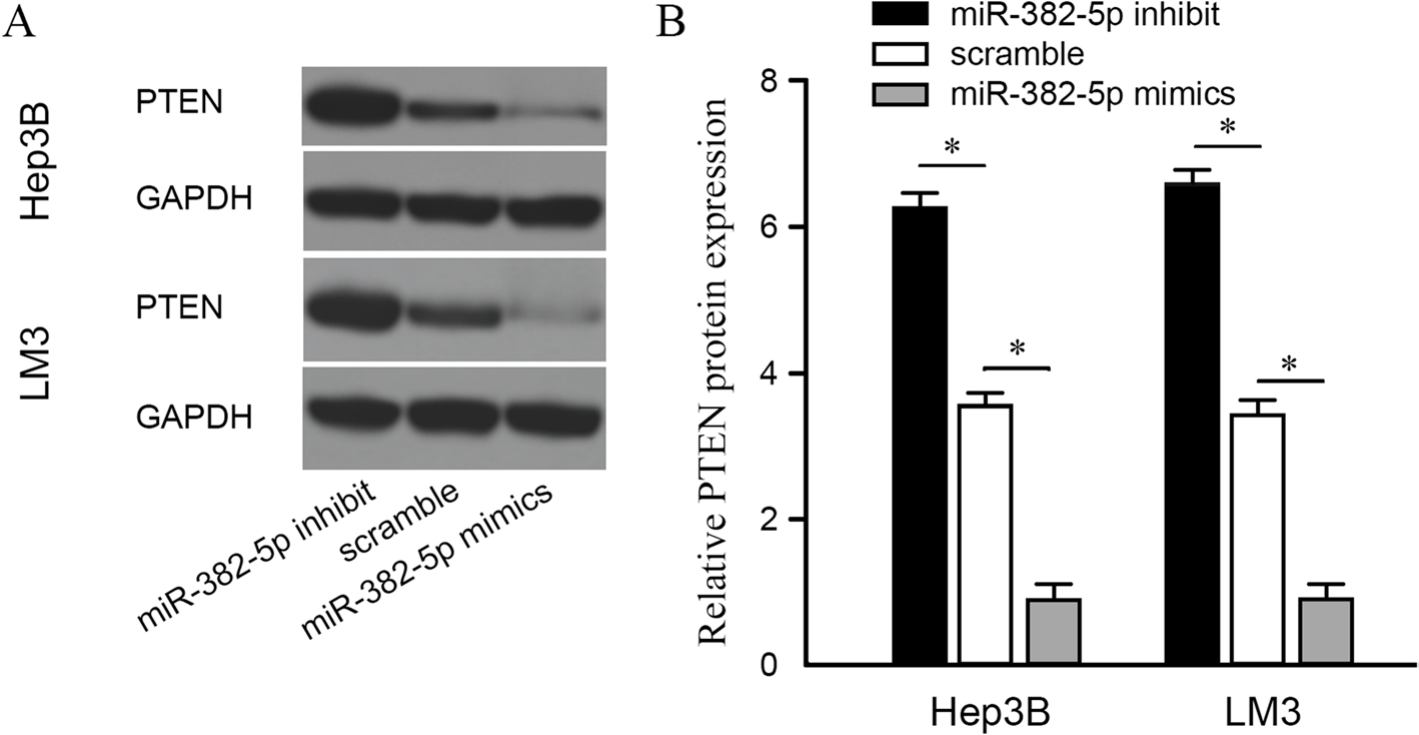
Targeted regulatory relationship between Mir-382-5P and PTEN in HCC cell lines. **A** PTEN expression of miR-382-5p mimic group, scramble group, and miR-382-5p inhibit group showed by Western blot. **B** Comparison of PTEN expression of miR-382-5p mimics group, scramble group, and miR-382-5p inhibit group; *P <0.05

PTEN expression in miR-382-5p mimics, scramble, and miR-382-5p inhibit were detected by Western blot. PTEN expression was lower in the miR-382-5p mimics group than that in the scramble group (Fig. 5A). Conversely, PTEN expression was higher in the miR-382-5p inhibit group than that in the scramble group (Fig. 5B). The above results showed that PTEN is a direct target of miR-382-5p, which means that miR-382-5p has a targeted regulatory relationship with PTEN, combining with duo-luciferase reporter gene assay.

### PTEN-mediated miR-382-5p promoted invasion of HCC cell lines (Hep3B and HCCLM3)

**Fig. 6:**
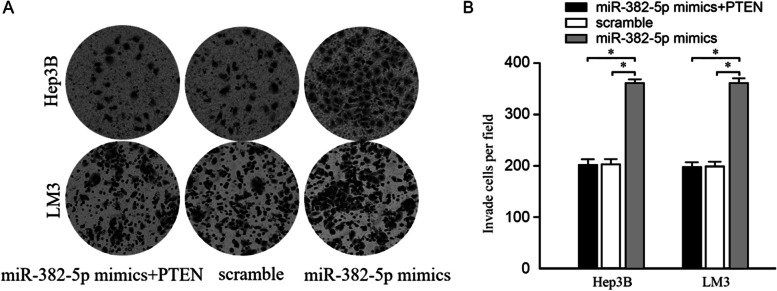
PTEN-mediated miR-382-5p promotes invasion of HCC cell lines (Hep3B and HCCLM3). **A** Transwell experiment showed that transfection of PTEN plasmid can impair the facilitation of miR-382-5p overexpression on the cell invasion of HCC cell lines; **B** Comparison of the number of invaded cells; *P <0.05

The rescue assay was applied to elucidate whether PTEN promoted miR-382-5p on HCC cell invasion. In this study, we transfected PTEN overexpression plasmids in Hep3B and HCCLM3 cell lines of overexpressed miR-382-5p. Transwell analysis showed that there were more invaded cells in the miR-382-5p mimics group than that in the scramble group, suggesting that the upregulation of miR-382-5p promoted Hep3B and HCCLM3 cell invasion. Transfection of PTEN overexpression plasmids can weaken the promoting effect of miR-382-5p overexpression on Hep3B and HCCLM3 cell invasion, while the number of invaded cells was not significantly different from the scramble group, as shown in Fig. 6.

### Effect of miR-382-5p expression on PI3K/Akt signaling pathway

**Fig. 7:**
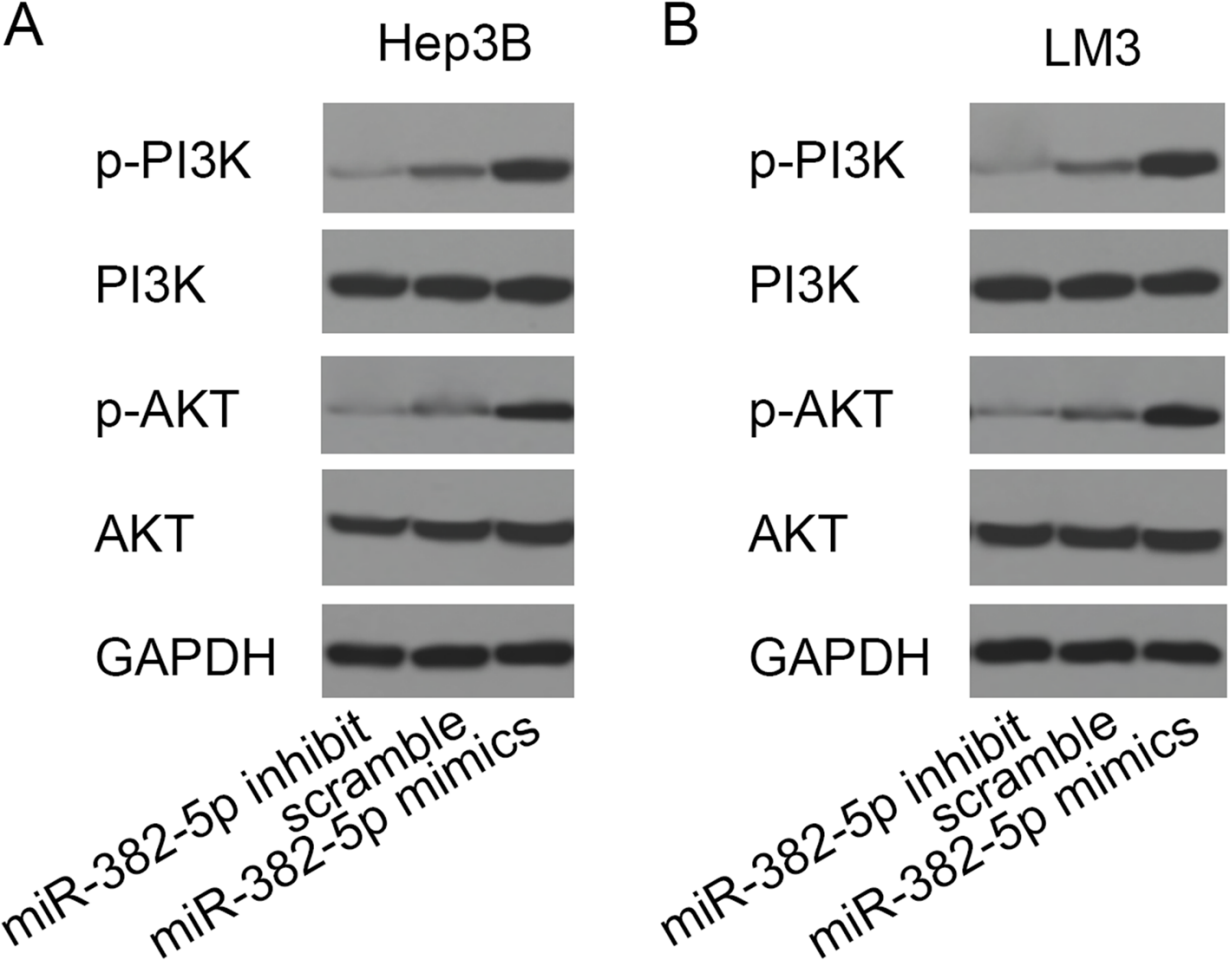
Effect of miR-382-5p expression on PI3K/Akt signaling pathway in Hep3B and HCCLM3 cell lines; **A** Western blot for changes of PI3K/Akt signaling pathway in Hep3B; **B** Western blot for changes of PI3K/Akt signaling pathway in HCCLM3

Given that PTEN is a major negative regulator of PI3K/Akt signaling pathway, this study explored whether miR-382-5p can alter the activity of PI3K/Akt signaling pathway in HCC cell lines. Western blot results showed that the expression levels of P-PI3K and P-Akt in Hep3B and HCCLM3 cell lines increased after transfection with miR-382-5P mimics, decreased after transfection with miR-382-5P inhibit, and did not significantly change after transfection of miR-382-5p mimics and miR-382-5p inhibit, indicating that miR-382-5p can activate PI3K/Akt signaling pathway, as shown in Fig. 7.

## Discussion

HCC, a common malignancy in the digestive system, is the third most common cause of cancer-related death in the world [17]. Patients with early HCC can receive surgery, while traditional chemotherapy has limited effects on HCC treatment due to drug resistance [18]. Further investigation of molecular mechanisms for the occurrence and progression of HCC is conducive to the discovery of new therapeutic targets and the improvement of prognosis. miRNA can be involved in the growth, differentiation, programmed death and immune processes of cells, and large numbers of miRNA are closely related to the development and progression of cancers, as important regulatory molecules [19]. A lot of evidence showed that dysregulation of miRNA can promote the growth and metastasis of HCC cells [20]. Therefore, the identification of HCC-specific miRNA and its targets is essential for understanding its role in the progression of HCC [21].

Previous studies have reported the role of miR-382-5p in various tumors. Xiaobo NIE [22] has reported that miR-382-5p is highly expressed in liver cancer tissues. miR-382-5p promotes the progression of HCC in vitro by suppressing FXR and could serve as a valuable therapeutic target for HCC treatment. Juan Du [23] reported that HBc-positive HCC tissues showed high miR-382-5p level and low DLC-1 expression. HBc promoted HCC motility by regulating the miR-382-5p/DLC-1 axis, which might provide a novel target for clinical diagnosis and treatment.

In this study, miR-382-5p expression in HCC tissues and adjacent tissues was analyzed by PCR, and the results showed that miR-382-5p expression was significantly upregulated in HCC tissues. And it was further found in cultured HCC cell lines in vitro that miR-382-5p expression was also significantly higher than that in normal hepatic cell lines. The above results suggest that miR-382-5p expression was upregulated in both HCC tissues and cell lines, which are consistent with the miR-382-5p expression in breast cancer [11] and acute lymphocytic leukemia [12]. It is consistent with the results reported by Xiaobo NIE [22] and Juan Du [23].

In this study, we further confirmed in HCC cell lines (Hep3B and HCCLM3) that upregulation of miR-382-5p was closely related to HCC cell invasion, respectively. Hep3B and HCCLM3 cells were transfected with miR-382-5p mimics and miR-382-5p inhibit to upregulate and downregulate the miR-382-5p expression, respectively. Then, the invasion ability of HCC cell lines cultured in vitro was significantly enhanced and decreased compared with the control group. It was suggested that mir-382-5P overexpression can promote the invasion of HCC cell lines.

TargetScan and dual-luciferase reporter gene experiments predicted and confirmed that PTEN is the target gene of miR-382-5p. It was also found that the expression level of PTEN protein was significantly lower in the cancer tissue of liver cancer patients than that in the adjacent tissue, and miR-382-5p and PTEN were significantly negatively correlated in the cancer tissue of liver cancer patients. Meanwhile, PTEN protein was downregulated after upregulation of miR-382-5p expression in hepatocyte lines (Hep3B and LM3) and vice versa. The above results confirmed that miR-382-5p could target and regulate PTEN protein expression. Given that PTEN is a common tumor suppressor gene in many tumors, and PTEN is closely related to the occurrence and progression of liver cancer, and low expression of PTEN can significantly promote the occurrence and progression of liver cancer [24–26]. The above findings suggested that miR-382-5p may be a potential therapeutic target for hepatocellular carcinoma, the targeted inhibition of miR-382-5p expression may have an inhibitory effect on hepatocellular carcinoma [27].

Upregulation of miR-382-5p expression in hepatic cell lines (Hep3B and HCCLM3) showed an increase in the number of invaded cells in HCC, while after co-transfection of miR-382-5P and PTEN plasmids, the invasion ability of HCC cells was weakened, and the number of invaded cells was not significantly different from that of the scramble group. These results suggest that upregulation of PTEN can alleviate the promoting effect of miR-382-5P on the invasion of HCC cells, indicating that miR-382-5P can exert its invasion on HCC cell lines to regulate PTEN by targeting.

Abnormalities in the phosphatidylinositol 3-kinase/serine-threonine kinase (PI3K/AKT) signaling pathway are strongly associated with tumor growth, maintenance, and chemotherapeutic resistance [28], which exhibits hyperactivation in HCC, and thereby promotes cell proliferation and invasion in cancers [29]. In this study, we found the potential target gene of miR-382-5p as PTEN. In previous studies, it was confirmed as a major negative regulator of PI3K/Akt pathway as PTEN [30], which can inhibit the activation of PI3K/Akt signaling pathway in HCC cells, thus inhibiting the progression of HCC. Recent studies have shown that upregulation of microRNA-17-5p activates PI3K/Akt signaling pathway to promote the invasion and metastasis of HCC cells by targeting and downregulating PTEN [31]. The latest studies have shown that downregulation of miR-188-5p can inhibit the activation of PTEN/PI3K/AKT signaling pathway, as well as hepatic fibrosis and the formation of adiposis hepatica [32]. In this study, the mechanism by which miR-382-5p promotes the invasion of liver cancer cells may be related to the activation of PI3K/Akt signaling pathway by the targeted regulation of PTEN.

## Conclusions

In conclusion, after upregulating miR-382-5p in the tissues and cell lines with HCC, miR-382-5p can activate PI3K/Akt signaling pathway by the targeted regulation of PTEN. It may become a new molecular target for liver cancer treatment.

## Data Availability

The datasets used and/or analyzed during the present study are available from the corresponding author on reasonable request.

## Abbreviations

HCC: Hepatocellular carcinoma
PTEN: Phosphatase and tensin homolog
miRNA: MicroRNA
